# Detection in blood of autoantibodies to tumour antigens as a case-finding method in lung cancer using the EarlyCDT®-Lung Test (ECLS): study protocol for a randomized controlled trial

**DOI:** 10.1186/s12885-017-3175-y

**Published:** 2017-03-11

**Authors:** F. M. Sullivan, Eoghan Farmer, Frances S. Mair, Shaun Treweek, Denise Kendrick, Cathy Jackson, Chris Robertson, Andrew Briggs, Colin McCowan, Laura Bedford, Ben Young, Kavita Vedhara, Stephanie Gallant, Roberta Littleford, John Robertson, Herb Sewell, Alistair Dorward, Joseph Sarvesvaran, Stuart Schembri

**Affiliations:** 1 Gordon F. Cheesbrough Research Chair & Director of UTOPIAN, Department of Family and Community Medicine University of Toronto, North York General Hospital, 4001 Leslie St LE140, Toronto, ON M2K 1E1 Canada; 2 School of Medicine,, St Andrews University, St Andrews, UK; 3 General Practice and Primary Care, Institute of Health and Wellbeing, University of Glasgow, Glasgow, UK; 4 Health Services Research Unit, University of Aberdeen, Aberdeen, UK; 5 School of Medicine, Division of Primary Care, Floor 13, Tower Building, University Park, Nottingham, UK; 6 School of Medicine, University of Central Lancashire, Preston, UK; 7 Department of Mathematics and Statistics, Livingstone Tower, 26 Richmond Street, Glasgow, G1 1XH UK; 8 Health Economics & Health Technology Assessment, Institute of Health & Wellbeing, University of Glasgow, Glasgow, UK; 9 Robertson Centre for Biostatistics, University of Glasgow, Glasgow, UK; 10 School of Medicine, Division of Primary Care, Medical School, Queen’s Medical Centre, Nottingham, UK; 11 Clinical Trial Manager, Tayside Clinical Trials Unit, University of Dundee, Dundee, UK; 12 Senior Clinical Trial Manager, Tayside Clinical Trials Unit, University of Dundee, Dundee, UK; 13 Graduate Entry Medicine & Health School (GEMS), University of Nottingham, Royal Derby Hospital, Nottingham, UK; 14 Division of Immunology, School of Life Sciences, Queens Medical Centre, Nottingham, UK; 15 Consultant Physician, NHS Greater Glasgow & Clyde, Glasgow, UK; 16 The Queen Elizabeth University Hospital Glasgow, 1345 Govan Road, Glasgow, G51 4TF UK; 17 Consultant Respiratory Physician, Ninewells Hospital, Dundee, UK

**Keywords:** Lung cancer, Early diagnosis, Screening, Health economics, RCT, Primary care, Biomarker, Autoantibodies

## Abstract

**Background:**

Lung cancer is the most common cause of cancer related death worldwide. The majority of cases are detected at a late stage when prognosis is poor. The EarlyCDT®-Lung Test detects autoantibodies to abnormal cell surface proteins in the earliest stages of the disease which may allow tumour detection at an earlier stage thus altering prognosis.

The primary research question is: Does using the EarlyCDT®-Lung Test to identify those at high risk of lung cancer, followed by X-ray and computed tomography (CT) scanning, reduce the incidence of patients with late-stage lung cancer (III & IV) or unclassified presentation (U) at diagnosis, compared to standard practice?

**Methods:**

A randomised controlled trial of 12 000 participants in areas of Scotland targeting general practices serving patients in the most deprived quintile of the Scottish Index of Multiple Deprivation. Adults aged 50–75 who are at high risk of lung cancer and healthy enough to undergo potentially curative therapy (Performance Status 0–2) are eligible to participate. The intervention is the EarlyCDT®-Lung Test, followed by X-ray and CT in those with a positive result. The comparator is standard clinical practice in the UK. The primary outcome is the difference, after 24 months, between the rates of patients with stage III, IV or unclassified lung cancer at diagnosis. The secondary outcomes include: all-cause mortality; disease specific mortality; a range of morbidity outcomes; cost-effectiveness and measures examining the psychological and behavioural consequences of screening.

Participants with a positive test result but for whom the CT scan does not lead to a lung cancer diagnosis will be offered 6 monthly thoracic CTs for 24 months. An initial chest X-ray will be used to determine the speed and the need for contrast in the first screening CT. Participants who are found to have lung cancer will be followed-up to assess both time to diagnosis and stage of disease at diagnosis.

**Discussion:**

The study will determine the clinical and cost effectiveness of EarlyCDT®-Lung Test for early lung cancer detection and assess its suitability for a large-scale, accredited screening service. The study will also assess the potential psychological and behavioural harms arising from false positive or false negative results, as well as the potential benefits to patients of true negative EarlyCDT lung test results. A cost-effectiveness model of lung cancer screening based on the results of the EarlyCDT Lung Test study will be developed.

**Trial registration:**

NCT01925625. August 19, 2013

**Electronic supplementary material:**

The online version of this article (doi:10.1186/s12885-017-3175-y) contains supplementary material, which is available to authorized users.

## Background

Lung cancer is the world’s leading cause of cancer related mortality and a major source of morbidity [1]. It is often diagnosed at an advanced stage with 85% of patients undiagnosed until the disease is symptomatic [2]. Scotland has one of the highest rates of lung cancer in the world [3]. Around 2 460 men and 2 340 women are diagnosed with lung cancer in Scotland every year, which is 16% of the total UK cases, despite Scotland having only 8% of the UK’s population. Survival from lung cancer is poor with less than 9% of patients still alive at 5 years after diagnosis, due primarily to the late stage of presentation [4]. Early detection and diagnosis of cancer improves prognosis - the current 5-years survival rate is approximately 60% for stage I lung cancer but is only 1% for those with stage IV disease [5].

The first studies evaluating screening for lung cancer utilised chest X-ray and/or sputum cytology [6–9]. While these showed increased numbers of earlier-stage, resectable cancers and improved survival rates in the screened groups, not all studies were randomised. The lack of trial strength data means that differences in lung cancer mortality between those screened and those not are difficult to interpret.

The National Cancer Institute National Lung Screening Trial (NLST) reported that CT screening reduced lung cancer mortality by 20% [10]. This has led to a number of guidelines in the United States which advocate lung cancer screening with low dose CT [11]. However as a primary screening modality CT is expensive and leads to a significant percentage of false positives (>90% of nodules are found to be benign) [12]. There was a substantial increase in morbidity associated with further investigation. More recently the UK Lung Cancer Screening Trial reported successful early detection of lung cancer using low dose CT scans [13].

The EarlyCDT®-Lung Test is a novel Autoantibody(AAB) diagnostic test for the early detection of lung cancer allowing stratification of individuals according to their risk of developing lung cancer [14]. This could permit a targeted approach to CT scanning for early lung cancer detection which may be a more cost-effective and potentially less harmful approach to population screening.

The EarlyCDT®-Lung Test measures seven AABs; p53, NY-ESO-1, CAGE, GBU4-5, HuD, MAGE A4 & SOX2. It identifies 41% of lung cancers with a high specificity of 90% [14]. This compares to CT scanning, which when used alone as a prevalence screening test, identifies 67% of lung cancers developing over the following 12 months, but has a low specificity of around 49% [10]. The autoantibodies detected in the test have not been shown to vary with age, gender and ethnicity [15].

In a large group of patients (*n* = 3 376) with newly diagnosed lung cancers there was no difference in positivity rate for the test in early or late stage disease lung cancers, and this applied to all lung cancers [14, 16]. Thus, while autoantibodies are present in early stage they are not simply a biomarker of early stage disease. While preliminary data shows promise there is insufficient evidence, as yet, to support the introduction of this test for cancer screening or a case finding program.

Consequently, the primary research question is: ‘Does using the EarlyCDT®-Lung Test, followed by X-ray and CT scanning, to identify those at high risk of lung cancer reduce the incidence of patients with late-stage lung cancer (III & IV) or unclassified presentation (U) at diagnosis, compared to standard clinical practice?

### AIMS

To assess the effectiveness of the test in increasing early stage lung cancer detection, thereby reducing the rate of late stage (III/IV/U) presentation compared to standard practice; to assess the cost-effectiveness of the test compared to standard practice; to assess the impact of the test on quality of life, positive and negative affect, illness perceptions, lung cancer risk perception, health anxiety, lung cancer worry, subjective stress related to screening, smoking behaviour and health service use.

## Methods

### Design

This is a randomised controlled trial involving 12,000 participants recruited through primary care and community based recruitment strategies in Scotland. < h3 > Setting.

General practices who serve patients in the lowest quintile of deprivation in Scotland, as measured by the Scottish Index of Multiple Deprivation, will be targeted [17]. Additional recruitment will be attained through adverts, posters, flyers and community based interactions and may extend to other practices as needed to ensure reaching our recruitment targets. Potential participants can either be seen at their participating GP practice or at the local clinical research centre, or other appropriate clinical location.

### Participants

Adults aged 50–75 who have at least a 2% risk of developing lung cancer over the next 24 months will be eligible to participate [18]. These are defined as those who are, current or former cigarette smokers with at least 20 pack-years, or have a history of cigarette smoking less than 20 pack-years plus an immediate family history (mother, father, brother, sister, child) of lung cancer which gives an individual a personal risk similar to a smoking history of 20 pack years. Participants should be healthy enough to undergo radical treatment either by pulmonary resection or stereotactic radiotherapy.

#### Number of participants

We will recruit 12,000 participants, from approximately 170 general practices.

#### Inclusion criteria

1. Participant is willing and able to give informed consent for participation in the study
2. Male or female aged 50–75 years
3. Current or Ex-smoker with at least 20 years pack history
4. Less than 20 years pack history but with family history of lung cancer in a 1st degree relative (mother, father, sister, brother, child)
5. Eastern Co-operative Oncology Group Status: 0, 1 and 2 [19]

#### Exclusion criteria

1. History of any cancer other than non-melanomatous skin cancer and/or cervical cancer in situ.
2. Complaining of symptoms suggestive of lung cancer within past 6 months i.e. haemoptysis or weight loss.
3. Patients for whom the GP considers invitation to the study would cause undue distress.
4. Patients with terminal disease.
5. Patients on prolonged/continuous use (>3 months) of Cyclophosphamide.

#### Randomisation

Participants will be allocated to the intervention or comparison group during the recruitment visit (Visit 1) using a web-based randomisation system TRuST [20]. Randomisation will be stratified by site and minimised by age, sex and smoking history.

### Dates and duration of trial

01/08/2013–31/07/18 (60 months).

#### Identifying participants

Practices in the most deprived areas will be approached by facilitators in the Scottish Primary Care Research Network (SPCRN) to participate. Potentially eligible individuals will be identified from GP medical records by an electronic medical record search [21]. Potential participants will be recruited via their General Practitioner and a range of other methods as recommended by the pre-trial focus groups [22]:

- postal invitation letter including a summary of the study Participant Information Sheet and a full Participant Information Sheet or Participant Information Brochure for those interested;
- invitation letter including a summary of the study Participant Information Sheet on collection of repeat prescription;
- invitation during consultation with GP/Practice Nurse/Health Care Assistant at the practice;
- invitation to those eligible on registered research volunteer databases
- poster present in the GP’s waiting room
- media campaign involving:

- ◦ local and national newspaper
- ◦ radio
- ◦ celebrity endorsement
- ◦ publicity campaign using posters/leaflets

The study invitation letter will include a slip for participants to either express interest in finding out more about the study [23]. Those returning an expression of interest will be telephoned, more than 24 h after anticipated receipt of the Participant Information Sheet, by a member of the research team. The call will allow a discussion of the study, to answer any questions the potential participant may have, do a preliminary assessment of eligibility and if agreed, to make an appointment for a recruitment visit. An appointment letter/email will be sent out to confirm appointment. A reminder call/email or text, whichever is preferable to the participant, will be carried our 2 days prior to the screening appointment to reduce non-attendance [24]. Non-responders to the postal invite will be contacted by letter again once or via a message on the right side of a repeat prescription [25]. Those returning an expression of interest will be sent a full information sheet and dealt with as above.

#### Initial consultation

The following procedures will be undertaken in the order given below:

- obtain consent
- take bloods from all consented participants
- complete study questionnaire
- randomise to treatment arm

### Administration of the test

After randomisation, all participants will be asked if they still wish to take part in the trial and still agree for their bloods to be used for the test and for future cancer related research. For participants randomised to the intervention arm the EarlyCDT®-Lung test will be performed and patients followed up according to their result (see Additional file 1: study flowchart).

At the initial visit, participants are told that those with a positive EarlyCDT®-Lung Test result will be invited to a follow-up visit to discuss the test results and explain what happens next. Those with a negative EarlyCDT®-Lung Test result will receive a letter explaining the test results and will be offered a follow-up visit or a telephone call if they wish. They will be told that the best way to reduce risk of developing lung cancer is by stopping smoking and that symptoms to watch for include persistent cough, coughing up blood, shortness of breath, weight loss or loss of appetite.

Those in the control arm will be written to and thanked for their contribution to the study and advised and counselled identically to those in the intervention arm who have had a negative EarlyCDT®-Lung Test result.

A patient specific section of the study website (www.eclsstudy.org) containing Participant Information Sheets and research staff contact details will be available for participants.

### Management of the visits

Based on the test’s reported 90% specificity and 41% sensitivity we anticipate that 520–550 participants in the intervention arm will have a positive test result. These will be offered a chest X-ray in accordance with local requirements for prioritisation and will be referred for a non-contrast thoracic CT scan. If there is a suspicious opacity on the chest X-ray or initial CT scan a contrast enhanced staging CT will be undertaken. As a quality control measure no participant undergoing CT screening in the test positive arm will have all their 5 CTs reported by same radiologist. Nodule size will be currently reported as the mean of 2 diameters at 90° angles, volumetric analysis is starting soon on both sites with diameter and volume to be reported. If the initial CT scan reveals no evidence of lung cancer then subsequent CT scans will be offered 6 monthly for 24 months. An appointment window of ± 4 weeks will be initiated for each scheduled CT scan.

If a test positive participant has had a chest X-ray in the previous 1 month, or a CT scan in the previous 3 months, these can be reviewed as part of the study. With the participant’s consent chest X-rays or CT scans prior to study entry will be retrospectively coded. The participant will proceed to have the series of up to 5 CTs.

Participants will receive appointments via post/email, according to patient preference. Participants will be called 2–4 days before each CT scan appointment. Individuals with abnormalities as classified by the radiology/respiratory physician’s study panel on baseline CT scan or subsequent CT scan will be followed up over the study period or referred for NHS clinical care as appropriate. All individuals entering the study will be flagged and followed-up via the Scottish Cancer Registry in the Electronic Data Research and Innovation Service (eDRIS) [26]. Participants who develop lung cancer will be followed-up via their medical records to assess both time to diagnosis and stage of disease at diagnosis. If no histological stage is available, stage will be assessed by a panel of three respiratory physicians blind to allocation status of the study subjects from chest X-rays or CT, or if no imaging is available, medical assessment of stage will be carried out.

Prior to sending CT scan appointment dates, the Scottish Community Health Index national register will be checked for vital status. All participants in the test- Positive test groups known to have died will be removed from the CT scan appointment schedule register. If patients (positive test) fail to attend for any imaging assessment during the study, they will receive two reminders (one letter, one phone call). On the third non-attendance, a letter will be sent to the participant’s GP to inform them of non-attendance.

Participants will receive results letters in relation to their initial chest X-ray and CT scan and subsequent CT scans. Any clinical intervention/treatment will be arranged by the relevant NHS multidisciplinary team.

### Control

The comparator is UK standard clinical practice which involves awaiting the development of symptoms and investigation of those symptoms according to national guidelines [27, 28].

### Intervention

EarlyCDT®-Lung Test blood sample followed by X-ray and serial cross sectional CT imaging in those with a positive result 6 monthly for 24 months. Those with a negative test, like the controls, have no further investigations but are provided with standard clinical care.

### Outcomes

#### Primary

The difference, at 24 months after randomisation, between the rates of patients with stage III, IV or unclassified lung cancer at diagnosis in the intervention arm, and those in the control arm;

#### Secondary

1. numbers at 24 months after randomisation, in the different stages at diagnosis (III/IV/U/other) in the intervention arm and the control arm;
2. difference, after 24 months, between costs and outcomes between the intervention arm and in the control arm and cost-effectiveness of the test compared to standard practice;
3. differences, after 24 months, of lung cancer mortality, all-cause mortality and cancer-specific mortality rates between the intervention arm and in the control arm;
4. differences, after 5 and 10 years, of long-term future mortality rates in the intervention arm and in the control arm;
5. differences, after 24 months in (i) the number of patients with stage III, IV or unclassified lung cancer at diagnosis in the test-positive group and those in the test-negative group and (ii) stage at diagnosis in the test-positive and test-negative group;
6. difference between the test-positive, test-negative groups and the control arm at 1, 3, 6, and 12 months in scores for EQ5D [29], Positive and Negative Affect Schedule [30], revised Illness Perception Questionnaire adapted to refer to lung cancer and lung cancer risk [31], Lung cancer risk perception, Health anxiety subscale of Health Orientation Scale [32], the Adapted Lung Cancer Worry Scale [33] and Impact of Events Scale [34] (for the test-positive group, the test-negative group only) and differences in smoking behaviour and health service use. Long-term scores for the same outcomes for the test-positive group at 18 and 24 months;
7. difference in incidence at 24 months, and after 5 and 10 years, in other clinical measures such as Cerebrovascular disease, Chronic Obstructive Pulmonary Disease, hospital stays, and outcomes identified through the Scottish Morbidity Record (SMR) linkage in the intervention arm and in the control arm
8. numbers in all groups at 24 months (test-positive, test-negative and control) undertaking subsequent investigations such as chest X-ray, CT and bronchoscopy (Table 1)

**Table 1:** Data Collection Timeline

Assessment/Procedures	Timeline (± 2 weeks)				
	Visit 1 (~30-45mins)		Visit 2 (~30mns)	➢ *EarlyCDT Positive Test Participants may visit or call.* ➢ *EARLY CDT Negative Test Participants may attend for further information/advice only.*	
*Informed Consent*	X				
*Inclusion/Exclusion Criteria*	X				
• *Review/Record only Relevant Medical History relating to IC/EC*	X				
• *Review/Record Relevant Medications* • *Relating to IC/EC*	X				
*Blood Sample*	X				
*Baseline Questionnaire*	X				
*Thank you letter to Control Group*		X			
*EarlyCDT- Lung Test Result Letter*		X			
*GP Results Letter & ICF copy (negative)*		X			
*Result Discussion/ Imaging Schedule*			X		
*Provide PIS 2*			X		
*GP Result Letter & ICF copy (positive)*			X		
EarlyCDT – Lung Test Positive Result Participants – Imaging Schedule					
	Timeline(± 12 weeks)				
	0	6 months	12 months	18 months	24 months
*CXR*	X				
*CT Scan*	X	X	X	X	X
*Scheduled every 6 months, if participant enters NHS clinical care pathway, subsequent study CT scans will be cancelled.*		*Research team member will call 2–4 days before each scheduled CT scan to check health status and attendance.*

### Statistics and data analysis

#### Sample size calculations

##### Main study

The rate of lung cancer was 187/100,000 per year for patients aged 50–74 in Scotland 2008 which is higher than many other similar countries [23]. Deprivation is associated with a significantly higher risk of lung cancer. Living in the most deprived quintile is associated with an increased risk of 1.8 times compared to the middle quintile of deprivation; this gives an estimated annual lung cancer rate of 336/100,000 among the practices taking part in the study. A high risk group within this population will be selected using similar entry criteria (outlined above) as the Mayo screening study which had a 2% prevalence rate of lung cancer and a further 2% incidence rate over the following 5 years [35]. The baseline rate of late stage presentation for the particular high risk population envisaged in this study is uncertain, as is the size of the reduction in late stage presentation likely to be achieved through use of EarlyCDT®-Lung Test. Using an estimated late stage presentation rate of 1,200/100,000 per year in the control arm i.e. 2.4% over the 2-years follow-up period, provides 85% power at 5% significance (two-sided) to detect an estimated reduction of 35% in late stage presentation rate in the intervention arm i.e. as low as 780/100,000 per year or 1.56% over the 2-years follow-up period. This corresponds to an estimated event rate over the 24 months of follow-up of 120 events in the control arm and 78 events in the intervention arm and implies a required sample size of 5,000 per arm i.e. a total of 10,000 participants.

The anticipated 35% reduction in event rate between the control arm and the intervention arm was justified by current estimates of the capability of the test to identify cases together with current estimates of the sensitivity of CT scanning (67%). The assumed event rate in the study participants of 1.2% per year was an estimate and the sample size would be modified if the observed event rate proves to be markedly different, acknowledging the a priori possibility that we will employ a prospective adaptive design. No Interim analysis of efficacy is planned.

The sample size calculations are based upon standard methods for time to event data using the c power function in R and st power exponential procedure in Stata and assuming exponential survival [34, 36]. They were also confirmed using standard approaches for detecting a change in binomial probabilities, and confirmed using approaches to detect a change in Poisson rates (with essentially identical results as loss to follow up is expected to be low due to completeness of Scottish Morbidity Register data).

The study aims for a short recruitment period and so no allowance has been made for accrual. With such an allowance, say to 1 year, the power will increase to 91% to identify a 35% reduction provided the minimum follow up period of 2 years is observed.

The initial assumptions of the rate of late stage presentation rate of 1,200/100,000 per year among the study participants was too optimistic and in January to May 2015 investigations were carried out to inform an increase in the sample size. Baseline information on the 8639 participants recruited to March 2015 (18 months from first randomisation) was used to derive an estimate of lung cancer risk based upon the Spitz Model. A number of variables in this model were not recorded in the study data base and low risk values were used in the risk calculation implying that the risk estimates should be underestimates. This suggested that the with 10,000 participants the rate of lung cancer would be expected to be around 680/100,000 and 540/100,000 for stage T3/T4/Unknown lung cancer using ISD cancer statistics figures of 80% lung cancers in Scotland are late stage. A sensitivity analysis around the missing data assumptions suggests that a late stage rate of around 600/100,000 may not be unreasonable, though is likely to be at the upper limit.

Using an assumption of 600/100,000 for late stage lung cancer, increasing the sample size to 12,000 [37], and acknowledging that recruitment is over a 2 years period the study has a power of 80% to detect a 35% reduction associated with the use of the EarlyCDT-Lung test to identify cases, provided that analysis takes place after all randomised patients have been followed up for 2 years. While an 80% power is at the lower end of acceptable powers this is the power level which has been used in a number of lung cancer screening trials.

The power of the study is sensitive to the assumptions about the rate of late stage cancer and the recruitment rate. A power in excess of 90% could only realistically be achieved by recruiting 15,000 patients or by changing the primary endpoint to 3 years post randomisation for all patients. If the recruitment phase extends past 2–2.5 years to recruit 12,000 participants then the power will increase slightly to 83%.

##### Substudies

For the follow-up analysis of behavioral and psychological outcomes, 200 participants in each group (test-positive, test-negative and the control arm) will allow detection of a mean difference of 3.00 (SD 15.04 (unpublished data comparing pre and post prostate biopsy scores from the ProtecT prostate cancer study)) in the Impact of Events Scale between baseline and follow up measurements. (http://www.ncbi.nlm.nih.gov/pubmed/21047592) This study reported within each group and a mean difference of 4.2 (SD 15.04), between each of the test groups and the control arm with 80% power and 2-sided 5% significance level. Assuming 80% of participants are current smokers, this will provide 80% power at 5% significance level to detect a 13% point difference in the prevalence of smoking between each of the test groups and the control arm (i.e. from 80 to 67%) To allow for attrition, we will recruit 300 participants in each group.

### Proposed analyses

Characteristics of participants will be compared informally between treatment arms at baseline. The main analysis of the primary outcome will be intention-to-treat. Cox proportional hazards models which will be used to estimate the hazard ratio of the rate of late stage lung cancer in the intervention arm compared to the control arm. Participants who are lost to follow up will be censored. The models will adjust for age, gender smoking history, socioeconomic status and practice. If appropriate, random cluster effects will be included rather than fixed effects for practices. A similar methodology will be used for the secondary outcomes of comparisons of mortality rates. A subsequent analysis will compare the outcomes of those with a positive test in comparison to those in the intervention group with a negative test (primary contrast for this analysis) and those in the control group. Comparisons of proportions will be carried out using chi square tests. Fisher’s exact test will be used if the number of events is small.

Psychological and behavioral outcomes will be compared between the three groups (Test-positive, Test-negative and the control group) at baseline using analysis of variance (or non-parametric tests if there is evidence of non-normal distribution of scores) for continuous measures and *χ* ^2^ tests for categorical measures. Psychological (HADS Score) and behavioral measures will be described at each follow up time point and multilevel regression models will be used for analyses to take account of repeated measurements during follow up [38].

Poisson regression models, adjusting for follow up time if necessary, will be used to investigate the other clinical measures (secondary outcomes 7 and 8).

### Cost effectiveness analysis

A short-term within-trial analysis will compare the costs and outcomes associated with the intervention arm to those of the comparison arm at 24 months, with a focus on cost-per-case detected. A longer term analysis will employ a decision analytic model to link the short term outcomes measured within the trial to potential longer term impacts on health, in terms of impacts on morbidity and mortality of early detection and treatment, to allow the estimation of cost-per Quality Adjusted Life Years gained. Both analyses will take the perspective of the NHS and personal social services and conform to the reference case favoured by NICE [39].

### Missing data

The extent of missing data will be examined and, if necessary, methods such as multiple imputation will be implemented to assess the robustness of results to missing data, assuming data are missing at random.

#### End of study

The end of study is defined as last patient last visit test-related scan plus 24 months. The Sponsor, CI and/or the Trial Steering Committee have the right at any time to terminate the study for clinical or administrative reasons.

### Data collection & management

#### Data collection

All research blood samples will be transported to the University of Nottingham for processing, and then transported to the US for Test processing by Oncimmune. All samples will be stored under custodianship as per UK Biobank guidelines [40]. Sample Analysis and Chain of Custody Plans are documented in the Study Operations Manual. The participant’s medical notes (GP and hospital) paper or electronic will act as source data for relevant past medical history, subsequent medical conditions, hospital admissions and diagnostic reports.

Psychological and behavioural data will be collected on the first 10,000 participants through a baseline questionnaire administered during Visit 1. Follow-up data will be collected between 1 and 12 months on subsets of the intervention and control arms and at 18 and 24 months for the EarlyCDT®-Lung Test-positive group. Data collected at baseline will include the EQ5D, Hospital Anxiety and Depression Scale, Positive and Negative Affect Schedule, revised Illness Perception Questionnaire adapted to refer to lung cancer and lung cancer risk, lung cancer risk perception, items from the Health Orientation Scale, the adapted Lung Cancer Worry Scale, smoking behaviour and demographic details. Follow-up questionnaires include the same measures, plus health service use and Impact of Events Scale and health service use for those who had the test. The Hospital Anxiety and Depression Scale is not included in follow-up questionnaires and the EQ-5D is not included in the 3 months follow-up questionnaire.

All participants in the positive group will be approached with the recruitment aim of 300 from this group. The TCTU will use an electronic randomisation tool to randomly sample patients from the test-negative group and control arm, stratified by the two study centres. Twenty-one individuals will be sampled each week and invited to complete follow-up questionnaires with an aim of recruiting 300 from both groups (based on an anticipated response rate of 67%).

Participants who receive a diagnosis of lung cancer will not be followed up subsequent to receiving the diagnosis.

### Data management and data management system

Data will be collected by the RN either directly onto a paper CRF with subsequent transcription to the eCRF, or direct data entry onto the web based eCRF.

TCTU will provide a data management system using OpenClinica [41]. The data management system will be fully validated, including the provision of test data and supporting documentation. Backup and disaster recovery will be provided by TCTU according to its standard operating procedures [42].

The Statistical Analysis Plan will specify dummy tables linked to primary and secondary outcomes and the data management system will be designed to export directly to the dummy table formats for analysis.

#### Safety assessments

Adverse Events (AE) and Serious Adverse Events (SAE) will be recorded. A number of factors affecting the trial population suggest that we would expect to observe a larger than normal incidence of episodes of ill-health due to both the age and co-morbidities of the study population. All chest X-ray and CT scan incidental findings will be recorded in the CRF as an incidental finding and a specialist referral will be made as directed in a study Standard Operating Procedure (SOP) within the Study Operations Manual [43]. AEs (as defined) will be recorded as soon as they are known either from the study subjects, PI patient review audits or via SMR or record review.

### Ethical considerations

The study will be conducted in accordance with the principles of good clinical practice (GCP) and the Research Governance Framework Scotland [44].

### Confidentiality

All records will be kept in a secure storage area with limited access to study staff only. Clinical information will not be released without the written permission of the participant, except as necessary for monitoring and auditing by the Sponsor, its designee or Regulatory Authorities.

#### Data protection

The CI and study staff involved with this study will comply with the requirements of the Data Protection Act 1998 with regard to the collection, storage, processing and disclosure of personal information and will uphold the Act’s core principles. The CI and study staff will also adhere to the current version of the NHS Scotland Code of Practice on Protecting Patient Confidentiality and all other governance requirements. Published results will not contain any personal data that could allow identification of individual participants.

#### Insurance and indemnity

The University of Dundee and Tayside Health Board are Co-Sponsoring the study.

## Discussion

Despite advances in surgical techniques, radiation therapy and systemic therapy the outlook for patients with lung cancer has improved more slowly than many other cancers over the last 50 years. Early diagnosis of treatable disease is likely to be the major way of changing outcomes for the foreseeable future. The study will assess the EarlyCDT®-Lung Test’s clinical suitability and cost effectiveness for a large-scale, accredited screening service for early lung cancer detection. It will also assess potential morbidity arising from the test and potential psychological and behavioural harms and benefits of test results.

The major strength of this trial lies within its design. By being both randomised and controlled many of the inherent biases that affected many of the previous screening studies will be removed. Our trial will also be able to investigate the effect of either a positive or negative result on participants’ lifestyle decisions to explore whether a negative result reinforces harmful behaviour, such as smoking, or a positive result reduces harmful behaviour. To date, screening with low dose CT scanning does not appear to have a beneficial effect on smoking behaviour [38, 45–47]. As in many screening studies a potential weakness is that the study population may be different to the usual clinical populations in terms of age, smoking status and education. Our eligibility criteria ensure participants have a high risk of lung cancer over the subsequent 24 months and our findings should be generalisable to populations with a similar level of risk. Our focus on areas with high levels of deprivation for recruitment should help ensure our participants reflect the social gradient in lung cancer incidence and our collection of demographic data will enable us to compare the characteristics of our trial population with those at risk of lung cancer.

One potential weakness is that participants randomised to the control arm may change their behaviour to decrease their risk of lung cancer e.g. by stopping smoking in a way that they would not have done had they not been participating in our study. The lack of impact of screening using CT scanning on smoking behaviour suggests this may not occur to an important extent, but measurement of smoking behaviour at repeated follow up time points will enable us to quantify this and assess its impact on our findings.

### Trial status

Recruitment began the 7^th^ of August 2013.

## Additional file

Additional file 1: Figure schedule of enrolment, interventions, and assessments. (DOC 53 kb)

## Abbreviations

AAB: Autoantibody
AE: Adverse event
CI: Chief investigator
CNORIS: Clinical negligence and other risks scheme
CRF: Care report form
CT scan: Computerised tomography scan
EarlyCDT®-Lung Test: Early cancer detection test
ECLS: Study early cancer detection test - lung cancer Scotland study
eCRF: Electronic case report form
eDRIS: Electronic data research and innovation service
GCP: Good clinical practice
HIC: Health informatics centre
IATA: International Air Transport Association
ICF: Informed consent form
ISF: Investigator site file
NLST: National lung screening trial
PANAS: Positive and negative affect schedule
RN: Research nurse
SAE: Serious adverse event
SCR: Scottish cancer register
SMR: Scottish morbidity record
SOP: Standard operating procedure
TAA: Tumour derived/associated antigens
TASC: Tayside medical science centre
TCTU: Tayside clinical trials unit
TMF: Trial master file
