# Supplementary material for: Detection in blood of autoantibodies to tumour antigens as a case-finding method in lung cancer using the EarlyCDT®-Lung Test (ECLS): study protocol for a randomized controlled trial
Source: BMC Cancer. 2017 Mar 11;17:187. doi: 10.1186/s12885-017-3175-y (PMC5346215; doi:10.1186/s12885-017-3175-y)
Supplement: Additional file 1: — Figure schedule of enrolment, interventions, and assessments. (DOC 53 kb) [file 12885_2017_3175_MOESM1_ESM.doc]

Figure schedule of enrolment, interventions, and assessments.

|  |  | | | | | | | |
| --- | --- | --- | --- | --- | --- | --- | --- | --- |
|  | **Enrolment** | **Allocation** | **Close-out** | | | | | |
| **TIMEPOINT**** | ***-t_1_*** | **0** | ***6m*** | ***12m*** | ***18m*** | ***24m*** | ***End of study*** |  |
| **ENROLMENT:** |  |  |  |  |  |  |  |  |
| **Eligibility screen** | X |  |  |  |  |  |  |  |
| **Informed consent** | X |  |  |  |  |  |  |  |
| **Allocation** |  | X |  |  |  |  |  |  |
| **Thank you letter to Control Group** |  | X |  |  |  |  |  |  |
| **EarlyCDT- Lung Test Result Letter and discussion** |  | X |  |  |  |  |  |  |
| **GP sent Results & Informed provided with Informed consent copy** |  | X |  |  |  |  |  |  |
| **INTERVENTIONS:** |  |  |  |  |  |  |  |  |
| ***Early CDT Positive***  ***CXR*** |  | X |  |  |  |  |  |  |
| ***CT scan*** |  | X | X | X | X | X |  |  |
| ***Early CDT Negative*** |  |  |  |  |  |  |  |  |
| ***Usual care*** |  |  |  |  |  |  |  |  |
| **ASSESSMENTS:** |  |  |  |  |  |  |  |  |
| **Blood Sample** | X |  |  |  |  |  |  |  |
| **Baseline Questionnaire EQ5D,  Hospital Anxiety and Depression Scale,  Positive and Negative Affect Schedule Revised Illness Perception Questionnaire Lung cancer risk perception The adapted Lung Cancer Worry Scale Smoking behaviour and demographic details.** | X | X | X | X | X | X | X |  |
| **Stage of cancer at diagnosis** |  |  |  |  |  |  | X |  |
| **Health service costs based on attendances, admissions, procedures, investigations and treatments** |  |  |  |  |  |  | X |  |
| **Mortality** |  |  |  |  |  |  | X |  |

*Recommended content can be displayed using various schematic formats. See SPIRIT 2013 Explanation and Elaboration for examples from protocols.

**List specific timepoints in this row.
